# Evidence-Based Nursing Model in Interventional Thrombolysis for Acute Lower Extremity Arterial Embolism

**DOI:** 10.1155/2022/4488797

**Published:** 2022-05-25

**Authors:** Meijuan Yang, Lijiao Chen, Min Zhang, Xiaoling Huang, Wenjun Zhao, Hui Wang

**Affiliations:** Department of Vascular Surgery, Taizhou Hospital, Zhejiang Province, Taizhou 317000, China

## Abstract

Acute lower extremity arterial embolism (AE) is a serious clinical emergency, and, if not treated in time, it can easily lead to limb ischemia and necrosis and eventually facing amputation, which seriously damages patients' physical and mental health. In the past, the conventional drug thrombolytic therapy had slow and limited efficacy, and the best time for treatment is easily delayed, while arterial dissection and thrombectomy treatment, although fast, is traumatic and has many complications, which is not easily accepted by patients. The aim of this study was to investigate the value of evidence-based care model in the application of interventional thrombolysis for acute lower limb arterial embolism. Seventy-two patients with acute lower limb arterial embolism who underwent interventional thrombolysis treatment received by the Department of Vascular Surgery of our hospital from July 2016 to December 2021 were randomly divided into a control group (given conventional nursing services) and a quality group (given full quality nursing services) to compare the effect of nursing services in the two groups. The results showed that the postoperative psychological status of patients in the quality group was significantly better than that of patients in the control group (*P* < 0.05). The total incidence of postoperative adverse events and the total treatment efficiency of the quality group were better than those of the control group (*P* < 0.05). The efficacy of quality nursing care in patients with acute lower extremity arterial embolism is more desirable than conventional nursing care and is recommended. The site of vascular occlusion after bypass surgery can be clarified when angiography is performed after thrombolytic therapy, which can help secondary surgical intervention to prolong the time to patency. The efficacy of quality nursing care in patients with acute lower extremity arterial embolism is more desirable than that of conventional nursing care and is recommended.

## 1. Introduction

Acute lower limb extremity ischemia (ALLI) is one of the emergencies in vascular surgery with an incidence of 1–1.5 per 1000 per year. ALLI results in a dramatic reduction in blood supply to the lower extremity and requires immediate restoration of perfusion once diagnosed [1]. Acute ischemic symptoms appear within 2 weeks, while chronic limb ischemia is rich in collateral circulation, which is able to replace occluded vessels. ALLI threatens the survival of the limb due to the short duration of the disease and the inability to establish collateral circulation, leading to the death of the patient in severe cases. In order to ensure the survival of the limb, blood flow reconstruction should be performed as soon as possible to restore the limb perfusion [2]. There are many causes of ALLI, such as trauma, tumor, aortic coarctation, arterial spasm disease, sepsis, arteritis, and popliteal artery trapping syndrome. The main etiologies include arterial embolism (AE) and arterial thrombosis [3–6]. The probabilities of arterial embolism, thrombosis due to atherosclerosis, combined factors, and thrombosis due to stents and grafts have been reported to account for 46%, 24%, 20%, and 10% of AEs, respectively. AE is mostly due to cardiogenic emboli, mostly caused by atrial fibrillation; other causes include valvular disease, including postvalve replacement, left ventricular wall thrombosis after myocardial infarction, cardiac and aortic redundancy, and paradoxical emboli, with the most common site of embolism generally being the femoral artery [7]. Vascular embolism can also result from atheromatous plaque rupture, aneurysmal appendage thrombosis, and catheter implantation-related medical emboli; embolism due to popliteal aneurysms is not very common [8].

The main causes of thrombosis as another cause of AE are atherosclerosis, hypercoagulable state of blood, vascular stenting, and arterial bypass surgery. Progressive narrowing of atherosclerotic vessels, once severe, will lead to platelet thrombosis and acute arterial thrombosis. Unlike arterial embolism, arterial thrombosis is progressive and therefore the collateral circulation is more abundant and often less clinically symptomatic [9]. Thrombosis can also form when the blood is in a hypercoagulable state [10–12]. Hypercoagulable thrombi are usually found in small arterial vessels and are associated with malignancy, hyperviscosity, and low flow states. In addition, lower extremity ischemia can result when the true lumen of the aortic coarctation compresses the false lumen. AE is one of the most common conditions in vascular surgery emergencies and can jeopardize the survival and life of the patient's example limb. If AE is suspected based on history and physical examination, the patient should be treated immediately with systemic heparinization. A total dose of 80 U/kg to 150 U/kg is administered at a rate of 18 U/kg/h, resulting in a partial thromboplastin time of 2 to 2.5 times the standard [13]. Subsequently, the treatment plan is graded according to the degree of ischemia in the affected limb. In patients with ischemia grade III with severe motor paralysis, stiffness, and skin cyanosis, irreversible damage to the limb has occurred and the risk of ischemia-reperfusion is high, requiring amputation [14]. When ischemia is grade IIb, immediate hemodynamic reconstruction should be performed to restore blood perfusion as soon as possible [15]. For patients with ischemia grade I and ischemia grade IIa, there is relatively more time. The modalities for restoration of limb revascularization include surgical treatment and endovascular treatment [15–18]. The severity of the underlying vascular disease, duration of ischemia, complications, and anatomic characteristics of the arterial vasculature are key factors in determining the mode of revascularization [19].

In patients with acute lower extremity arterial embolism, femoral artery dissection balloon embolization is preferred. Thrombectomy with a thrombectomy catheter has changed the way AE is treated and improved the outcome. The earlier the treatment, the better the results of thrombectomy, because the early thrombus has not yet adhered to the arterial intima and is particularly suitable for patients with simple arterial embolism. The use of embolization catheters and improvements in clinical care have led to a significant decrease in AE mortality over the past decade or so. However, up to 30% of patients after balloon thrombectomy have thrombotic remnants associated with the angiographic procedure. In patients with peripheral vascular disease, thrombectomy is usually followed by reoperative endarterectomy. The aim of this study was to investigate the value of an evidence-based care model in the interventional thrombolysis of acute lower extremity arterial embolism. Seventy-two patients with acute lower limb arterial embolism who underwent interventional thrombolysis treatment received by the Department of Vascular Surgery of our hospital from July 2016 to December 2021 were randomly divided into a control group (given conventional nursing services) and a quality group (given full quality nursing services) to compare the effect of nursing services in the two groups. The results showed that the postoperative psychological status of patients in the quality group was significantly better than that of the patients in the control group (*P* < 0.05); the total incidence of postoperative adverse events and the total treatment efficiency of the quality group were better than those of the control group (*P* < 0.05).

## 2. Patient Information and Methods

### 2.1. Patient Information

Seventy-two patients with AE who underwent interventional thrombolysis from July 2016 to December 2021 were admitted to our vascular surgery department. Inclusion criteria were as follows: meeting the clinical diagnostic criteria of AE, confirmation by ultrasound and angiography of the lower limbs, meeting the indications for interventional thrombolysis, and voluntary participation in this study. Exclusion criteria were as follows: contraindication to interventional thrombolytic therapy, previous history of thrombolytic therapy, significant organ dysfunction, previous history of mental illness, and refusal to participate in the study. The 72 patients were divided into the control and quality groups according to the random number table method, and the differences in baseline data between the two groups were not statistically significant (*P* > 0.05) and were comparable, as shown in Table 1.

### 2.2. Care Methods

**Figure 1:**
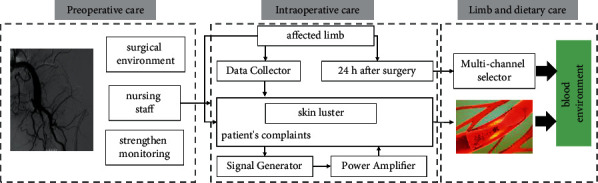
Evidence-based care model.

In the control group, patients were given conventional interventional thrombolytic therapy care, a detailed medical history before the operation, perfect preoperative examination and preparation, early rest in the evening 1 d before the operation to ensure a good mental state during the operation, close cooperation with the operation, postoperative monitoring of signs and symptoms, and conventional anticoagulation treatment as prescribed by the doctor. In the quality group, the entire quality care service was given on the basis of the control group, as shown in Figure 1. The purpose of systemic anticoagulation is to stop thrombus development and inhibit secondary thrombosis. Anticoagulation should be contraindicated in patients with active bleeding.

*Preoperative Care*. Patients with AE usually have anxiety and nervousness when they are admitted to the hospital because they lack sufficient knowledge about the disease and interventional thrombolytic therapy and have doubts about the effect of treatment. The nursing staff should patiently explain the pathogenesis of AE and the advantages and precautions of thrombolytic therapy to the patients before surgery and ensure the patients' right to know about the treatment. At the same time, it is necessary to enhance the patient's family support role to strengthen their treatment hope, so that the patient can meet the surgery with a happy attitude and improve the tolerance of the surgery. In addition, AE patients usually feel severe pain, which can be relieved by painkillers as prescribed by the doctor to improve arterial spasm, and take good measures to keep the limb warm, prohibiting warmth through hot water bags or hot water immersion to avoid aggravating the symptoms of the affected limb.

*Intraoperative Care*. Patients are easily fearful of the surgical environment during surgery, so nursing staff should accompany them, stabilize their emotions by touching and patting the back of their hands, divert their attention by talking, encourage them to relax, ask them about their feelings frequently, strengthen the monitoring of their signs, and handle any discomfort in a timely manner.

*Limb and Dietary Care*. Check the affected limb every 30 min for 24 h after surgery, observe the skin luster, temperature, and sensation of the affected limb, listen to the patient's complaints, understand whether there is any pain or abnormal sensation, and instruct the family members not to apply cold or hot compresses to the affected limb to avoid aggravating the condition due to vasoconstriction. In addition, the patient should be instructed to eat more fiber-rich foods and drink 1 cup of warm water every morning to promote defecation to avoid constipation and thrombosis and drink 1 cup of warm water before bedtime to thin the blood and improve the internal blood environment, as shown in Table 2.

**Figure 2:**
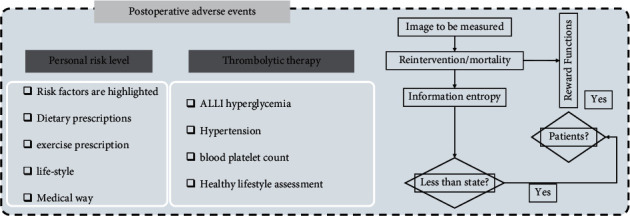
Management of postoperative adverse events.

The observation and care of postoperative adverse events are shown in Figure 2, and CDT can also be performed after arterial bypass surgery when a thrombus has formed in the bypass vessel. In a systematic study, post-CDT angiography after reocclusion of an artificial vessel showed a patency rate of 82%, and post-CDT angiography after occlusion of a bypass vessel with an autologous vein graft showed a patency rate of 61%. In a Swedish study, 123 patients with AE underwent CDT (67% received an artificial vessel graft) with a mean thrombolysis time of 19 hours; only 29% did not require adjunctive surgical intervention after thrombolysis, 21% underwent open surgery, 39% received endovascular treatment, and the 1-month and 1-year survival rates for patients without amputation were 89% and 75%.

Arteriotomy balloon thrombectomy or bypass surgery alone plays an important role in AE, but when patients have complex, varying degrees of occlusive disease, they are best treated with a hybrid surgical approach. Endovascular techniques such as intra-arterial thrombolysis or thrombus aspiration/mechanical thrombectomy can be used to remove residual thrombus if it is found after arterial thrombectomy. When angiography after thrombectomy reveals an underlying chronic stenotic lesion, arterial balloon angioplasty or stenting can also be used to treat the underlying lesion and prolong the patency time. Likewise, endovascular treatment requires open surgical assistance with endovascular surgery followed by endarterectomy or osteofasciectomy. Therefore, treatment of patients with AE should be performed in a hybridized operating room or an operating room with a C-arm and by a clinical team capable of performing both open and endovascular procedures. Although hybridization has been widely accepted, the superiority of hybridization in the treatment of AE has been less well reported. A recent multicenter retrospective study analyzed the recent outcomes of 1480 patients with AE treated with open surgery, endovascular surgery, and hybrid surgery.

However, the results of the study showed no difference in the rate of reintervention and mortality at 30 days after the procedure. In this study, two patients in the arteriotomy group underwent angiography for stenosis: one patient underwent endarterectomy and the other underwent bypass surgery with contralateral saphenous vein graft; in the CDT group, one patient underwent endarterectomy due to atherosclerosis, and the patient recovered well after surgery. A large, multicenter clinical trial is needed to further confirm whether endoluminal surgery is superior to open surgery in the treatment of AE.

1. Hemorrhage: Hemorrhage is the most common problem of interventional thrombolytic therapy, usually caused by poor vascular suture and overdose of anticoagulant drugs. To effectively avoid the occurrence of hemorrhage after thrombolytic therapy, the patient's consciousness should be closely observed after the operation, and the incision should be observed for blood oozing and swelling. Blood transfusion, rehydration, and hemostatic treatment should be given in a timely manner.
2. Pressure ulcers: Pressure ulcers are also a common problem in patients with AE after thrombolytic therapy. Patients should be assisted in turning regularly after surgery, keeping the hip joint on the puncture side straight, and placing a soft pillow behind the back to avoid direct contact between the affected limb and hard objects to avoid skin breakdown. Skin cream should be applied according to medical advice.
3. Organ embolism: To avoid organ embolism due to postoperative dislodgment of the embolus, the patient should be placed in a head-high, foot-low position and the head of the bed should be elevated by 30° after surgery.
4. Catheter care: Properly fix the catheter, record the depth of catheter placement in detail every day, and strictly disinfect the catheter and the tip of the sheath before injecting thrombolytic drugs to drain the air inside the catheter, and then seal the catheter with sodium heparin to avoid catheter blockage.

### 2.3. Efficacy Assessment

**Figure 3:**
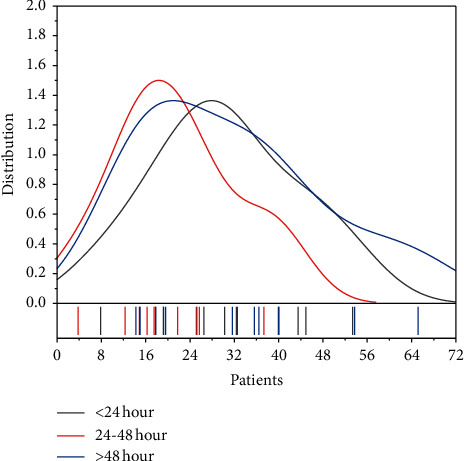
Change in thrombotic residual stenosis rate.

Patients were routinely monitored postoperatively with cardiac monitoring and bed rest (no flexion). The lower limb blood flow (lower limb skin temperature, skin color, femoral artery, popliteal artery, dorsalis pedis artery, and posterior tibial artery pulsation) and lower limb swelling (especially the gastrocnemius muscle) were closely observed, and the lungs were auscultated for wet rales and breath sounds. In the CDT group, the coagulation function and blood routine were reviewed daily, and fibrinogen was controlled to be greater than 1 g/L. The patients were also closely monitored for bleeding from skin and mucous membranes, gums, urine, and stool and for bleeding around the sheath. After 24–48 hours of thrombolysis, reangiography is performed to clarify the thrombolytic situation. When stenotic lesions are found on angiography, balloon angioplasty is feasible, and when the residual stenosis rate of thrombus is >30%, a vascular stent can be implanted. Stent placement is also indicated in patients with partial thrombosis of the arterial vessel wall after atherectomy, as shown in Figure 3. The risk of bleeding was higher with thrombolytic therapy, and the incidence of hemorrhagic cerebrovascular disease was 1.6%, with major bleeding accounting for (requiring blood transfusion) 13.2%.

**Figure 4:**
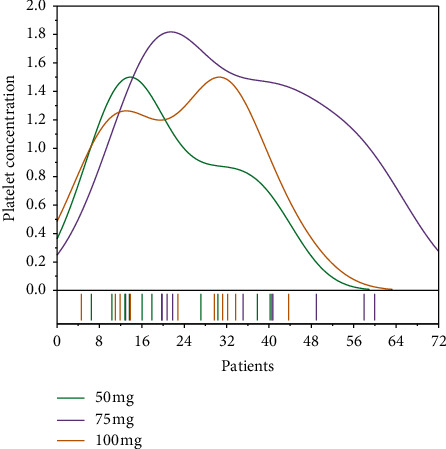
Changes in platelet concentration of dual antibodies.

In patients with lower extremity atherosclerotic thrombosis, thrombolysis is not effective and further balloon dilation or stent placement may be performed depending on the stenosis. Patients in both groups were given early postoperative hydration and alkalinization to reduce injury due to ischemia-reperfusion. Long-term postoperative Warfarin anticoagulant therapy is as follows: the long-term goal is that the international standardized ratio (INR) of all patients with AF or patients with embolism from other cardiac sources, artificial heart valves, or known hypercoagulable state is 2‐3. For thrombotic patients with peripheral vascular disease, dual antiplatelet (aspirin 100 mg once a day, clopidogrel bisulfate 75 mg once a day) is combined with anticoagulation for 3 months, followed by monoclonal antibody platelets (aspirin 100 mg once a day, or clopidogrel bisulfate 75 mg once a day) for 1 year to prevent restenosis, as shown in Figure 4.

The main risk of arterial thrombolysis is bleeding, approximately 13%–30%, with a small but usually fatal risk of cerebral hemorrhage (approximately 0.4%–2.3%) and 8%–10% of patients requiring transfusion therapy due to massive bleeding. After CDT, the most common complication is bleeding at the puncture site. To prevent bleeding, the sheath should be properly secured during thrombolytic therapy and coagulation should be closely monitored. Early minor bleeding can be prevented by applying direct hand pressure to the puncture site, resecuring the sheath, or replacing it with a larger vascular sheath to prevent rebleeding. If bleeding is more severe, urokinase pumping should be stopped. When limb ischemia is more severe, treat minor bleeding and continue to lyse the thrombus with a lower dose of urokinase to save the limb. In this study, the bleeding rate in patients in the CDT group (9.4%) was significantly higher than that in the surgical group (0%), with a statistically significant difference (*P* < 0.05).

The postoperative infection rate was higher in the open surgery group (11.4%) than in the CDT group, with a statistically significant difference (*P* < 0.05). There were no patients with bleeding in the incisional embolization group, and, in the CDT group, there were 3 cases of bleeding (9.4%), which were stopped by adjusting the urokinase dose and refixing the sheath. Thrombolytic therapy has a longer revascularization time compared with open surgery, and further worsening of limb ischemia may occur during treatment if thrombolysis is ineffective. Accurate assessment of the recovery of blood flow in the limb is extremely important for the prognosis, so angiography should be performed during thrombolysis to clarify the effect of thrombolysis according to the recovery of blood flow. If there are signs of further worsening of limb ischemia or if the symptoms of limb ischemia do not improve within 6–12 hours after the procedure, a change in treatment strategy should be considered. Fibrinogen is depleted during thrombolysis, and, to reduce the occurrence of bleeding, fibrinogen levels should be monitored and the dose of thrombolytic drugs should be regulated.

## 3. Results and Analysis

### 3.1. Treatment Effect

In both groups, 58 patients were successfully treated (healed and good), with a surgical success rate of 80.56%. 32 patients were healed and were good in the arteriotomy group, with a success rate of 72.7%, and 26 patients were healed and were good in the CDT group, with a success rate of 81.3%. The success rates of the two groups were not statistically significant (*P* > 0.05). There were 2 amputations and 4 deaths in the arterial dissection and embolization group, with an amputation rate of 4.5% and a mortality rate of 9.1%, and 1 amputation and 2 deaths in the CDT group, with an amputation rate of 3.1% and a mortality rate of 6.3%; and there were no statistically significant amputations and deaths in the dissection and embolization group and the CDT group (*P* > 0.05). The bleeding rate in the CDT group was higher than that in the dissection and embolization group (9.4% versus 0%), and the difference was statistically significant (*P* < 0.05). The infection rate was higher in the arterial dissection and embolization group than in the CDT group (11.4% versus 0%), and the difference was statistically significant (*P* < 0.05).

**Figure 5:**
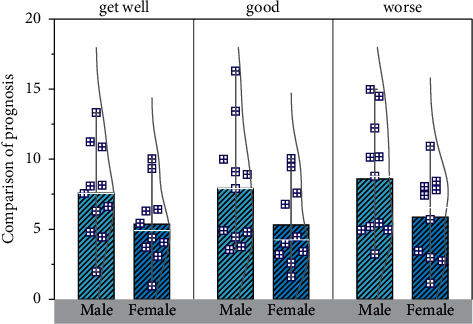
Comparison of prognosis between the ischemia time placement thrombolysis group and the incision and extraction group.

The duration of hospitalization was 11.23 ± 2.62 days in the incisional embolization group, 10.511 ± 3.69 days in the CDT group, and 3.36 ± 1.07 days in the thrombolysis group; the duration of hospitalization in the CDT group was less than that in the incisional embolization group, but the difference was not statistically significant (*P* > 0.05); patients were divided into three groups according to the ischemic time, namely, 0–6 h, 6–24 h, and >24 h, to analyze the prognosis of patients with lower extremity arterial embolism with different ischemic times under the two procedures, as shown in Figure 5.

**Figure 6:**
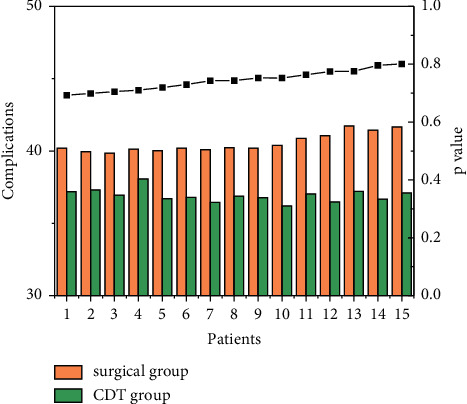
Comparison of complications between the surgical and CDT groups.

In this study, 30-day postoperative complications included bleeding, infection, pseudoaneurysm, lymphatic fistula, renal insufficiency/hyperkalemia, and osteofascial compartment syndrome, among which 5 cases of incisional infection occurred in the incisional group, which were treated with surgical anti-infection and clean dressing change, and the incisional site recovered well; 2 cases of lymphatic fistula occurred in the incisional group, which recovered in 1 case after dressing change and the other case had a poor prognosis; 3 cases of pseudoaneurysm occurred in the CDT group, which improved after adjustment of urokinase pumping rate. In the CDT group, 3 cases of subcutaneous and gingival bleeding and perisphincter bleeding improved after adjusting the pumping rate of urokinase; in the thrombolysis group, 1 case of pseudoaneurysm occurred and recovered well after surgical aneurysm resection; 7 cases of postoperative renal insufficiency and hyperkalemia improved after treatment with diuretic and potassium antagonist; 3 cases of femoral fascial compartment syndrome improved after treatment with incisional decompression and drug changes, as shown in Figure 6. The three cases of femoropopliteal compartment syndrome were treated by incision and decompression and drug replacement, as shown in Figure 6.

For patients undergoing open surgery, early prophylactic application of antibiotics and intraoperative aseptic operation should be performed in order to prevent surgical incision infection. Arteriotomy to remove the embolus can quickly restore the blood flow of the limb; however, the long-time ischemia of the lower limb and the rapid entry of a large amount of toxins into the blood after the opening of the vessel can lead to renal insufficiency and cardiovascular accidents. In the authors' opinion, the toxin-bearing blood can be released after opening the vessel and then the vessel can be sutured to reduce the toxin entry into the blood, but its safety and effectiveness are to be further verified in clinical trials. Slow restoration of blood circulation to the limb can control IRI and has been extensively studied. CDT surgery may reduce IRI due to the long time to restore limb circulation compared to arteriotomy embolization. An RCT trial showed that amputation and mortality rates at 4 weeks and 1 year were not significantly improved with conventional surgery and thrombolysis. Prophylactic fasciotomy is effective in preventing CS and apparently makes it easier for patients who have already undergone open surgery to undergo osteofascial compartment dissection. However, there were many confounding factors in this trial, including the timing of the osteotomy, making it difficult to draw firm conclusions from these data. Osteofasciotomy carries a risk of infection, and early skin grafting or other coverages of the wound may reduce the rate of infection.

It has also been shown that about half of the patients who undergo osteofasciotomy develop postoperative deep venous insufficiency, which becomes more pronounced over time. Therefore, the decision to perform an osteofasciotomy should be carefully considered. In this study, lymphatic fistula occurred in two patients in the arteriotomy and embolization group, and one patient recovered after a clean change of medication, while the prognosis of one patient was poor. For patients who underwent femoral artery puncture, to prevent the formation of hematoma and pseudoaneurysm, according to clinical experience, the puncture site is compressed and bandaged with direct hand compression for half an hour, and the dorsalis pedis artery pulsation should be touched during compression to avoid limb ischemia. Seven cases of postoperative acute renal insufficiency and hyperkalemia improved after treatment with alkalinization, hydration, diuresis, and antagonism of potassium ions. Three patients with postoperative CS resolved after treatment with osteofascial compartment dissection and clean drug changes. Early recognition of patients with CS is particularly important, and, once diagnosed, early incision and decompression should be performed to prevent further worsening of ischemia.

### 3.2. Discussion of Results

**Figure 7:**
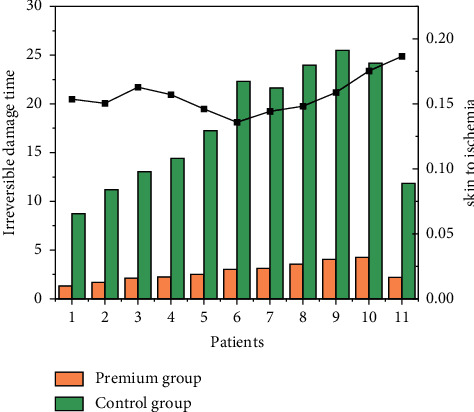
Irreversible damage time of nerve tissue, muscle tissue, and skin to ischemia.

AE is a disease that causes acute ischemia and hypoxia in the limbs due to sudden interruption of arterial perfusion from various causes. The main etiologies are arterial embolism and atherosclerotic thrombosis, which account for 46% and 24% of AE, respectively. The typical clinical manifestations of acute lower extremity arterial embolism are the “6P” signs: limb pain, skin pallor, pulselessness, abnormal sensation, limb chills, and paralysis. Acute lower extremity arterial embolism progresses rapidly, and delayed diagnosis and treatment may lead to amputation or death. Different tissues in the body have different tolerance times for ischemia, with irreversible damage to nerve tissue, muscle tissue, and skin lasting 4–6 hours, 6–8 hours, and 8–12 hours, respectively, as shown in Figure 7. In general, patients with Rutherford ischemic grade I AE require urgent revascularization within 12 hours, while patients with Rutherford ischemic grade II AE require urgent revascularization within 2–6 hours, and patients with Rutherford ischemic grade III AE present with tissue necrosis and permanent nerve injury, and revascularization is not necessary. The blood flow reconstruction is not necessary. Restoration of blood perfusion can lead to organ dysfunction and death associated with ischemia-reperfusion, and amputation should be considered after initial stabilization. In the early stage of lower limb ischemia, with soft thrombotic mass and no secondary thrombosis, as well as low risk of IRI, in order to reduce amputation rate and morbidity and mortality, clinicians need to make timely recognition, receive early systemic heparinization therapy to stop thrombus development and inhibit secondary thrombosis, and restore limb perfusion in a timely manner in order to reduce mortality and lower amputation plane. The gold standard for the diagnosis of AE is lower extremity angiography, but there are problems such as long examination time, radiation risk, and renal impairment. Lower extremity vascular ultrasound is a noninvasive test that can rapidly diagnose AE and should be preferred. A recent study reported that patients with delayed fasciotomy were more likely to require amputation within 30 days than patients with prophylactic fasciotomy (50% versus 5.9%), suggesting that prophylactic fasciotomy is beneficial to patients.

Intraluminal and open surgical treatment of acute lower extremity arterial embolism has been extensively reported in the literature; however, there is still no consensus on the optimal treatment modality for acute lower extremity arterial embolism. Arterial thrombectomy, the primary treatment modality for acute lower extremity arterial embolism, has been reported in the literature in 82 patients (49%) with AF, in most cases using the transfemoral route (aortic, iliac, and femoral embolization), with 10 patients (6%) in this study undergoing postoperative rebypass surgery, 16% undergoing local thrombolysis, and 39% requiring osteofascial dissection. The 30-day mortality rate was 18%, and another 15% was amputated within 90 days, with a five-year survival rate of 80% for amputation.

**Figure 8:**
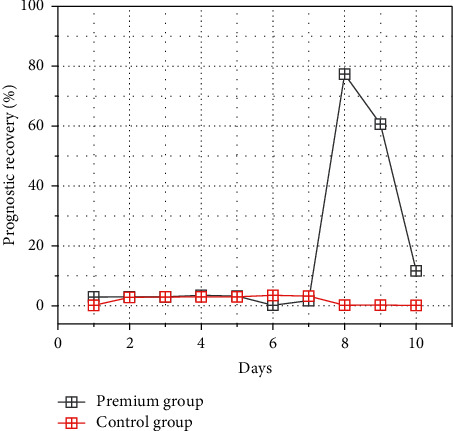
Prognostic recovery in patients with different ischemic times in the embolization and CDT groups.

In this study, the amputation rate (4.5% versus 3.1%) and mortality rate (9.1% versus 6.3) were higher in the incision and embolization group than in the CDT group, but none of the differences were statistically significant (*P* > 0.05). This may be due to the fact that patients with ischemia level IIb were higher in the incisional embolization group than in the CDT group (34.09% versus 28.13%). Usually angiography is performed after arteriotomy balloon thrombectomy, which allows identification of not only residual thrombus in the distal vessels but also peripheral vascular lesions. In a few cases, angiography may even reveal vascular injury due to balloon catheter use. This requires the surgeon to accurately assess the adequacy of intraoperative thrombus removal, which can be performed by angiography, angioscopy, or intravascular ultrasound. During arteriotomy for thrombus removal, the appropriate type of balloon should be selected as well as gentle handling during thrombus removal to reduce intimal damage. Open surgery is very traumatic, with long anesthesia, high thrombus residual rate, and problems such as high infection rate, lymphatic fistula, and long hospital stay. In this study, the hospital stay was 11.23 ± 2.62 days in the open embolization group and 10.51 ± 3.69 days in the CDT group, which was shorter than that in the open embolization group, but the difference was not statistically significant (*P* > 0.05); compared with surgical procedures, CDT is less invasive, has a shorter average hospital stay, and can reduce the occurrence of IRI. There was no significant difference in the prognosis between the arteriotomy and CDT groups at different ischemic times (*P* > 0.05), as shown in Figure 8. In patients with thrombosis combined with peripheral vascular lesions, there is often residual thrombus after dissection and thrombectomy, and CDT is recommended.

With the advancement of interventional techniques and patient demand for minimally invasive techniques, CDT has become an important treatment modality for acute lower extremity arterial embolism, with a lower amputation rate with endoluminal treatment than with open surgery (6.5% versus 13.5%). Studies have shown that acute lower extremity arterial embolism has a high mortality rate of approximately 10.1%, and endovascular procedures in this study had a lower mortality rate than surgical procedures (4.3% versus 13.0%). Recent literature reports that endovascular surgery significantly reduces 30-day mortality in patients undergoing open surgery compared to endovascular treatment. The 30-day mortality rate was higher in the surgical group compared to the endovascular group (12.1% versus 6.7%). However, another study showed no difference in length of stay and 1-year limb preservation rates between open and endoluminal surgeries, as well as similar 1-year amputation-free survival and short-term overall survival rates.

The endovascular technique had a higher 1-year amputation-free survival rate compared with surgery; however, confounding factors were not excluded in the STILE trial. Endovascular luminal procedures have a higher rate of reintervention compared with initial open surgery. Endovascular procedures have higher bleeding complications, increasing the incidence of cardiopulmonary complications, blood loss, wound infection, and fasciotomy in patients. CDT requires some time for thrombolysis to restore perfusion and may not be appropriate for advanced acute lower extremity arterial embolism requiring immediate revascularization. Endovascular procedures can improve limb preservation rates and are safer in the short term than emergency open revascularization. However, the choice of surgical approach for individual patients needs to be carefully considered to maximize patient benefit. Endovascular procedures and open procedures are complementary in the treatment of acute lower extremity arterial embolism, and long-term treatment outcomes need to be confirmed by additional high-quality clinical trial data.

## 4. Conclusion

In patients with acute lower extremity arterial embolism, femoral artery dissection balloon embolization is preferred. Thrombectomy with a thrombectomy catheter has changed the way AE is treated and improved the outcome. The earlier the treatment, the better the results of thrombectomy, because the early thrombus has not yet adhered to the arterial intima and is particularly suitable for patients with simple arterial embolism. Seventy-two patients with acute lower limb arterial embolism who underwent interventional thrombolysis treatment received by the Department of Vascular Surgery of our hospital from July 2016 to December 2021 were randomly divided into a control group (given conventional nursing services) and a quality group (given full quality nursing services) to compare the effect of nursing services in the two groups. The results showed that the postoperative psychological status of patients in the quality group was significantly better than that of the patients in the control group (*P* < 0.05); the total incidence of postoperative adverse events and the total treatment efficiency of the quality group were better than those of the control group (*P* < 0.05). The efficacy of quality nursing care in patients with acute lower extremity arterial embolism is more desirable than conventional nursing care and is recommended. In the future, the application of good nursing care will also play a key role in the prognosis of AE patients.

## Figures and Tables

**Table 1:** Comparison of the information of the two groups of patients.

Group	Number of patients	Gender (male/female)	Age	Length of hospitalization	Site of disease		
					Left lower extremity	Right lower extremity	Both lower extremities
Quality group	36	16/20	60.2 ± 0.23	12.2 ± 0.56	12	23	1
Control group	36	18/18	56.3 ± 0.12	14.2 ± 0.26	15	20	1

**Table 2:** Factors affecting exercise capacity.

Group	Quantity	Preoperative score		Postoperative score		
		Pressure sores	Organ necrosis	Shedding	Hemorrhage	Catheter blockage
Quality group	36	1 (2.23)	2 (1.25)	1 (1.23)	1	1
Control group	36	1 (2.63)	2 (2.78)	1 (1.36)	5	4

## Data Availability

The data used to support the findings of this study are available from the corresponding author upon request.
